# Performing Microdissection Testicular Sperm Extraction: Surgical Pearls from a High-Volume Infertility Center

**DOI:** 10.3390/jcm10194296

**Published:** 2021-09-22

**Authors:** Giovanni M. Colpi, Ettore Caroppo

**Affiliations:** 1 Andrology Unit, Procrea Institute, 6900 Lugano, Switzerland; 2 Asl Bari, PTA “F Jaia”, Andrology Outpatients Clinic, 70014 Conversano (Ba), Italy; ecaroppo@teseo.it

**Keywords:** microTESE, sperm retrieval, non-obstructive azoospermia, male infertility

## Abstract

Microdissection testicular sperm extraction (mTESE) has been demonstrated to be the gold-standard surgical technique for retrieving testicular sperm in patients with non-obstructive azoospermia (NOA) as it enables the exploration of the whole testicular parenchyma at a high magnification, allowing the identification of the rare dilated seminipherous tubules that may contain sperm, usually surrounded by thinner or atrophic tubules. MTESE requires a skilled and experienced surgeon whose learning curve may greatly affect the sperm retrieval rate, as demonstrated in previous reports. The present review is intended to offer a precise and detailed description of the mTESE surgical procedure, accompanied by an extensive iconography, to provide urologists with valuable information to be translated into clinical practice. Advice about the pre-surgical and post-surgical management of patients is also offered.

## 1. Introduction

Despite the lack of sperm in the ejaculate, patients with azoospermia due to spermatogenic dysfunction, the so-called non-obstructive azoospermia, may still be able to father children genetically on their own as residual, focal areas of spermatogenesis may be present in their testes. Due to the anatomical singularity of such a condition, often characterized by the heterogeneous distribution of histologically and functionally distinct seminiferous tubules (STs) [1], a randomly applied biopsy is able to retrieve sperm in about one third of the cases; on the other hand, by enabling the exploration of the whole testicular parenchyma, microdissection testicular sperm extraction (mTESE) is one point five times more successful than the conventional TESE (cTESE) [2] and is therefore considered the gold-standard surgical technique for patients with NOA. In addition, the exploration of the testicular parenchyma at a high magnification (24–36×) enables the retrieval of a significantly higher number of sperm to be used for intracytoplasmic sperm injection (ICSI) compared to that of cTESE.

The superiority of mTESE compared to other surgical techniques has been challenged by a more recent meta-analysis [3], whose results are, however, significantly affected by the heterogeneity of treated populations and reporting bias [4]. Esteves et al. re-evaluated the data of the meta-analysis on the basis of eligible controlled studies with histopathological data and found that the sperm retrieval rate (SRR) was 49% for mTESE vs. 35.8% for cTESE (RR 1.37 (1.14–1.65); *p* = 0.0004); for cases with Sertoli cell-only syndrome, SRR was 36.1% for mTESE vs. 13.3% for cTESE (RR 2.70 (1.72–4.24); *p* < 0.0001) [4].

Notably, when performing mTESE the skill and experience of the surgeon is key to a successful sperm retrieval. The sperm retrieval rate (SRR) of mTESE is strongly influenced by the surgeon’s case volume: Ishikawa et al. [5] showed that the SRR increased after the first 100 mTESEs performed, and Dabaja and Schlegel [6] observed that the SRR further increased when the surgeon exceeded experience with more than 500 mTESE procedures. The surgical experience accumulated in the past twenty-three years by our leading urologist (GMC) supports what is suggested by these authors: as displayed in Table 1, the mTESE sperm retrieval rates improved over time with the number of mTESE procedures.

The aim of the present narrative review is to share the surgical experience of an expert urologist by providing surgical tips and tricks in the management of patients with non-obstructive azoospermia.

## 2. Preoperative Patient Optimization

Spermatogenesis usually takes 74 days in humans. As physical (particularly occupational heat exposure) and lifestyle factors (recreational drug abuse, high fat diet, alcohol intake, etc.,) may compromise male reproductive health, it is advisable that men with NOA willing to undergo mTESE should be adequately counselled about those risks, which are more susceptible to undervaluation [7]. For men with known exposure, a three- to-six-month washout period may be advisable before proceeding with mTESE; those patients with previous surgical sperm retrieval should wait six months before undergoing a further surgical attempt.

Some patients with NOA may have a subclinical or clinical asymptomatic hypogonadism, in most cases due to primary testicular failure and in a few cases due to hypogonadotropic hypogonadism. As testosterone signaling is required for spermatogenesis to proceed beyond meiosis, it has been postulated that patients with hypogonadism should have their serum testosterone levels optimized before surgical sperm retrieval. Indeed, a pooled estimation of seven studies reporting the sperm retrieval rates in patients with subnormal vs. normal testosterone levels demonstrated that patients with normal testosterone levels had a significantly higher chance of successful sperm retrieval (SSR) compared to those with hypogonadism (OR 1.63, 95% CI 1.08–2.45, *p* = 0.02) [8]. The utility of the hormonal treatment of patients with NOA has been evaluated by a few studies, yet with conflicting results [8]. The recent AUA/ASRM guidelines on the diagnosis and management of male infertility recommend informing patients with NOA about the limited data supporting pharmacologic treatments prior to surgical intervention [9].

Performing scrotal ultrasound before surgery may provide valuable information for the surgeon. The testis volume should be evaluated by using the known formula of length × width × height × 0.52, while the ultrasonic texture may be evaluated according to Lenz et al. [10]. In addition, the testis ultrasound may reveal areas of fibrotic tissue, due to previous surgery or trauma, and the presence of testicular nodules, which may not be uncommon in men with NOA, given the significantly higher risk of testicular cancer in these patients compared to infertile men with less severe spermatogenic impairment (standardized incidence ratios—SIR 2.9, 95% CI 1.4–5.4), particularly in younger men (SIR 3.7, 95% CI 1.7–7.0) [11]. The risk of testicular cancer may be even higher in patients with cryptorchidism [12].

Patients with NOA with clinical varicocele may undergo varicocele repair before surgical sperm retrieval as it may result in the detection of ejaculated sperm or in better sperm retrieval rates [13]. Such a beneficial effect may be more effective for patients with histological evidence of hypospermatogenesis than for patients with maturation arrest or Sertoli cell-only syndrome [14]. Postponing mTESE to let patients undergo varicocele repair may, however, be not advisable for couples with female factor fertility (e.g., female age >38 years, poor ovarian reserve); as the potential benefits of varicocele repair are not obtained until at least 3–6 months after the repair, this would lead to unjustified delays in IVF treatments.

## 3. MTESE Procedure

The average duration of surgery, in our experience, is 87′ (range 60′–140′) for unilateral mTESE and 126′ (65–205′) for bilateral mTESE. MTESE is usually performed under general anesthesia; due to the inherent psychological stress, we try our best to avoid unneeded painful experiences for our patients by administering ketorolac 30 mg plus paracetamol 1 gr and pethidine 1 mg/kg one hour before awakening from anesthesia-induced unconsciousness. Ketorolac and paracetamol may be administered again eight hours later.

Generally, the larger testis is first chosen for the mTESE evaluation, apart from selected cases (the presence of testicular nodules or microlithiasis in the smaller testis at ultrasound or previous surgery on the larger testis). A 1.5–3 cm wide scrotal incision, performed in parallel to the skin vessels, ensures an almost invisible scar one month after surgery. Following the testis exposure, the tunica vaginalis is opened, then a 4–10× magnification allows the identification of a testicular surface area devoid of sub-albugineal vessels where an equatorial or para-equatorial incision can be safely made. In the case of a salvage mTESE after failed sperm retrieval attempts, the albugineal incision should be made far enough from the scars as the testicular tissue closer to the scars may be atrophic due to previous tissue excision and to vascular damage. Even testicular aspiration may inflict severe and irreversible damage to the testicular tissue and to the architecture of the tubules in the needle’s path, as demonstrated by an animal study [15]; for this reason, performing a salvage mTESE after a failed multifocal TEFNA may be more challenging than after a cTESE.

**Figure 1:**
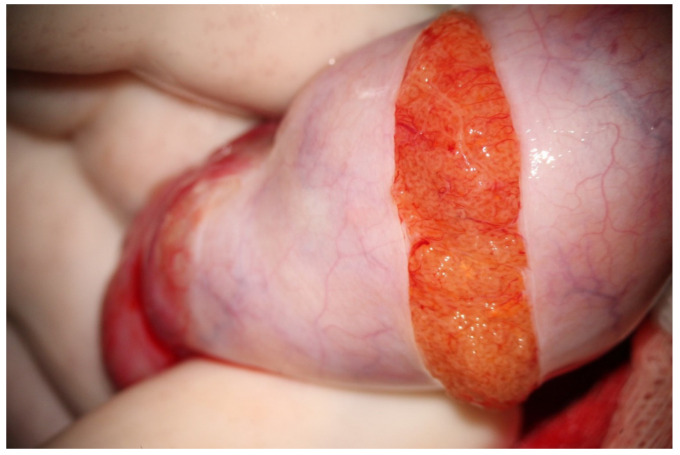
Wide incision of albuginea.

Following albugineal incision, which may cover 180 to 270° of the testicular circumference (Figure 1), the two albugineal edges are held by two mosquito clamps, and the testicular parenchyma is observed at high magnification (×36) while the surgeon holds the testis firmly to allow a correct evaluation of the parenchyma under the operating microscope. The careful and thorough search for areas containing those STs that appear clearly dilated compared to the surroundings represents the most important step of mTESE as it has been demonstrated that dilated STs may contain sperm in 90% of cases [16]. Indeed, the testicular parenchyma of patients with NOA is commonly made of tiny tubules, containing only Sertoli cells, or with complete hyalinization, while dilated STs may lie solitary, surrounded by smaller size tubules or grouped in tiny heaps or, more rarely, occupy a small part of a lobule. Less frequently, STs may appear homogeneously dilated; this typically occurs in cases of early or late maturation arrest. Patients with NOA due to maturation arrest usually have normal FSH levels and testicular volume; in these cases, it may be advisable to make a less wide scrotal incision as the whole testicular parenchyma may be homogeneously made of dilated STs, and a larger incision would not improve the sperm retrieval outcome.

**Figure 2:**
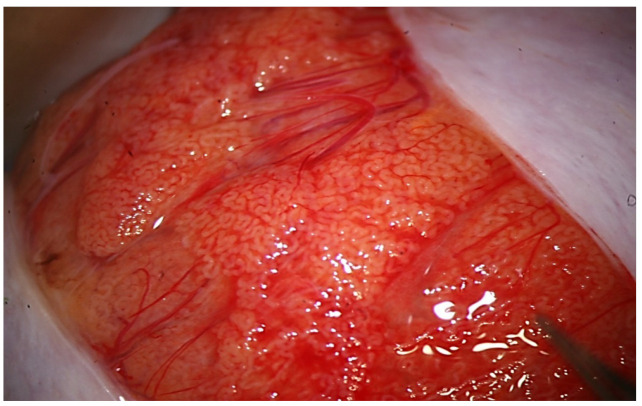
Testicular parenchyma at medium-high magnification.

During the exploration at high magnification, the testicular parenchyma is gently detached (Figure 2) to individuate dilated tubules, avoiding any traction that may distort the STs caliber. Hemostasis with a bipolar microcoagulator should be avoided at this point (in some cases it may be applied only to the intra- or sub-albugineal vessels) and eventually limited to a gentle pressure on the testicular tissue for 4 min using gauze wet with Ringer solution. In this phase a small fragment of testicular tissue, representative of the overall appearance of the testicular parenchyma, is taken, fixed in Bouin’s solution, and sent to the pathologist for histological examination. Testis histology is mandatory for classifying the predominant histological pattern and for excluding the presence of intraepithelial neoplasia [17], which is more common in patients with NOA compared to non-azoospermic infertile men [11]. In addition, a histopathological report may represent a cross-validation of the biological report: the presence of sperm in a previous histological section of a patient with sperm retrieval failure (e.g., Sertoli-only syndrome) may suggest the presence of focal areas of hypospermatogenesis that may justify a salvage mTESE.

**Figure 3:**
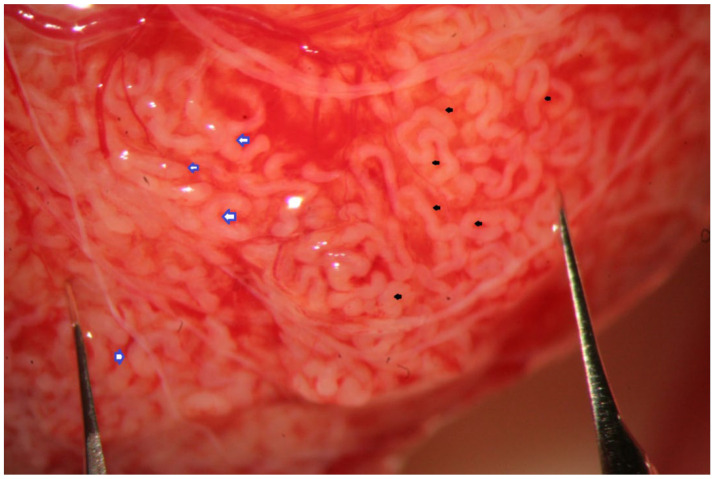
High magnification (×24- > ×36) allows the easy discrimination between seminiferous tubules of different sizes (those marked by blue arrows are very slightly larger than those marked by black arrows).

The evaluation of the testicular parenchyma at high magnification (×36) enables the surgeon to discriminate between STs that, at a lower magnification, may appear of comparable size (Figure 3).

**Figure 4:**
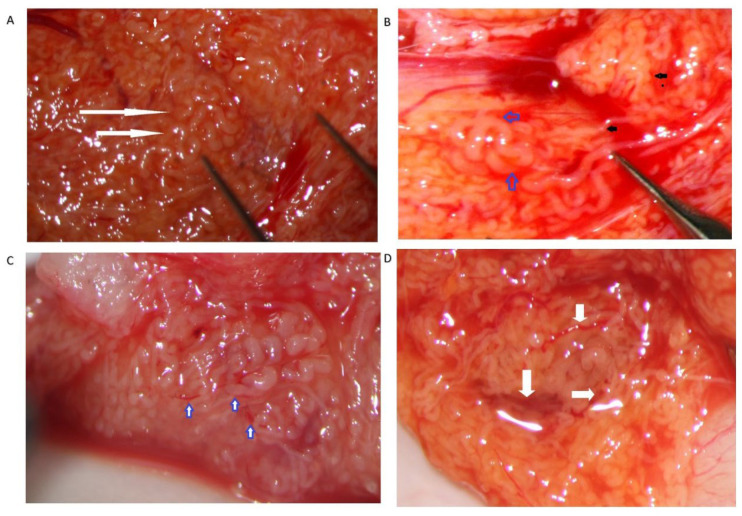
Dilated tubules. (**A**) Medium-sized group of dilated tubules (long arrows) among not-dilated tubules of different caliber. (**B**) Small group of dilated tubules (blue arrows) dispersed among non-dilated tubules (black arrows). (**C**) A small lobule of dilated tubules crossed and flanked by tiny blood vessels (arrows). (**D**) A group of large opaque tubules surrounded by blood vessels (arrows).

The STs of better caliber, which are often opaquer than the surroundings (Figure 4), are removed with Vannas micro-forceps, washed in human tubal fluid medium to remove the blood, and transferred to a sterile Petri dish containing Ham’s F10 medium with serum substitute supplement; the embryologist then minces them extensively until they can be passed through a 24-gauge angiocatheter. Then, a 1 mL collagenase solution is added to the fragments, and the samples are incubated at 37° for two hours. The resulting cellular suspension is diluted with a medium and centrifuged twice at 800× *g* for ten minutes, then the pellet is observed under a phase contrast microscope. To save time, the procedure may be performed by two embryologists working in parallel. In a few minutes, the embryologist may give a response about the presence of sperm in the suspension; if sperm are found, the surgeon proceeds with the identification of STs of the same caliber, removes those grouped together with Vannas micro-forceps, and brings them to the embryologist for a rough estimate of the number and quality of sperm retrieved. When the number of sperm retrieved is adequate for the ICSI, the surgeon stops the research for dilated STs.

**Figure 5:**
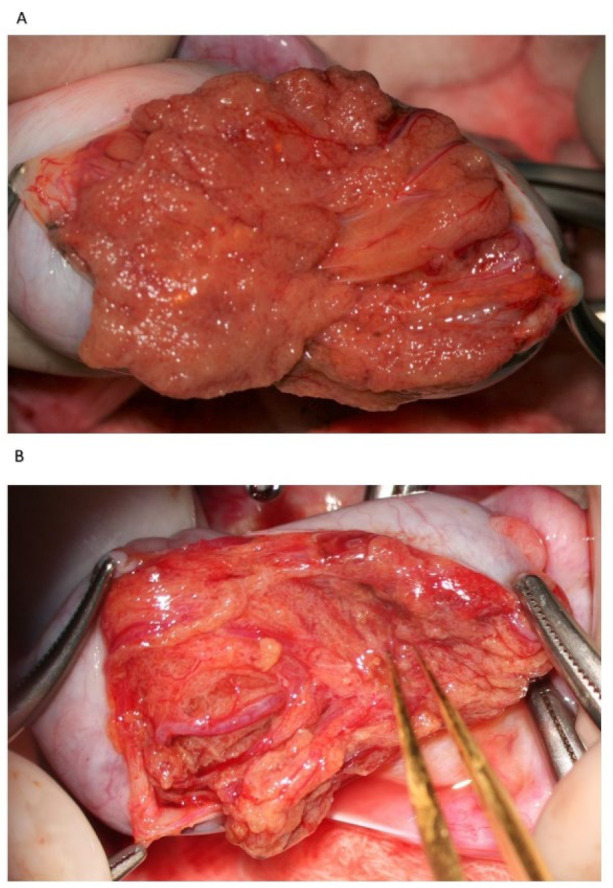
(**A**) Medium-degree bivalve testis opening; (**B**) large bivalve testis opening.

**Figure 6:**
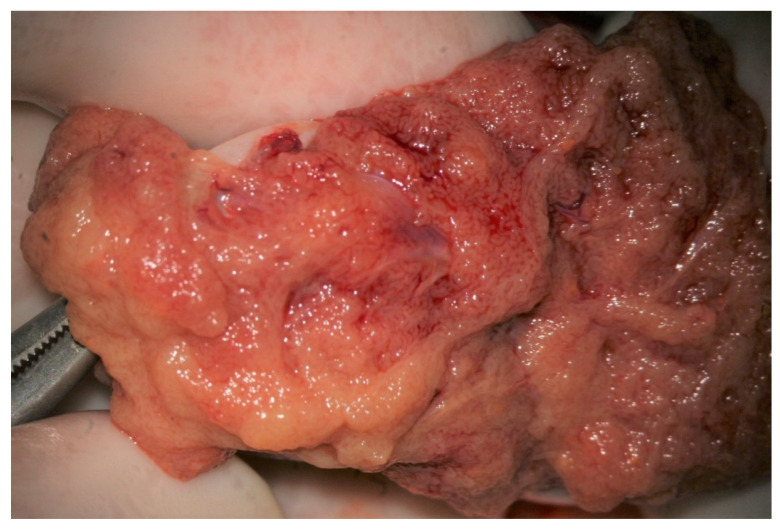
Extremely wide testis opening with exploration in the bi-polar direction and partial extrusion of lobules.

The number and quality of retrieved sperm, and their planned use in ICSI cycles (fresh or frozen), may affect the duration of surgery and the amount of tissue dissection. In the case of easily retrieved sperm, most of the testicular tissue is spared [18], which may represent an undoubted advantage in the case of a further salvage mTESE that could become necessary for further ICSI attempts. The search for sperm could be less extensive in the case of a fresh ICSI-mTESE as few viable sperm may be needed for the ICSI. For this reason, several authors prefer using freshly retrieved testicular sperm for the ICSI [19,20]; indeed, although no statistical difference has been demonstrated between the use of fresh versus cryopreserved-thawed testicular sperm with regard to fertilization and pregnancy rates in ICSI cycles [21], fresh ICSI-mTESE requires the yield of fewer sperm compared to the frozen ICSI-mTESE as only 33% of frozen-thawed testicular sperm will be viable for use with the ICSI [19]. Supernumerary testicular sperm should be frozen, obviously, for further use in the ICSI. Still, there are some drawbacks when using the fresh mTESE-ICSI, including the possibility of an otherwise unforeseeable sperm retrieval failure, with the consequent need for oocyte cryopreservation, as well as organizational issues. When the number of retrieved sperm is insufficient for the ICSI, the surgeon proceeds with a wider examination of the testicular parenchyma by a bivalve full opening (Figure 5). If needed, the deeper part of the testicular parenchyma is explored orthogonally to the para-equatorial section plan, avoiding as much as possible any possible vascular damage (Figure 6), and the tubules are examined both along the septa and by delicately detaching groups of them from the adjacent ones by Vannas micro-forceps. For large testes, if the initial incision does not adequately provide exposure to the entirety of the testicular parenchyma, a second parallel equatorial incision is performed. When no dilated STS are identified, any tubule whose caliber appears slightly larger than that of the surroundings is removed (the so-called slightly dilated tubules) [16]. If no sperm are found, then not-dilated tubules are excised according to a sort of mapping by removing tiny fragments of testicular tissue from the two separated surfaces at different depths from the albuginea to the hilum. In our experience, however, sperm are found in not-dilated tubules in only 7% of cases [16]. In the case of sperm retrieval failure, the contralateral testis is opened with the modality described above.

**Figure 7:**
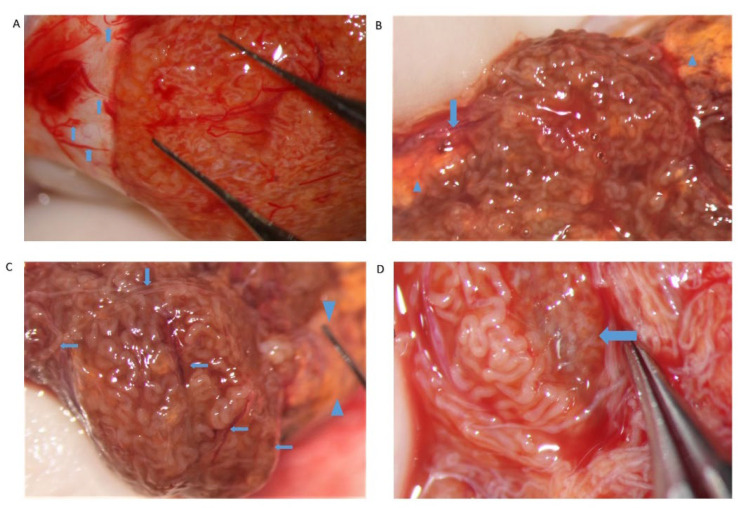
(**A**) Dilated tubules are found close to sub-albugineal vessels (arrows); (**B**) a lobule of dilated tubules supplied by a vessel (arrow), amidst two yellow areas full of Leydig cells (wedges); (**C**) an entire lobule of tubules with different calibers, but mainly dilated, well nourished by many vessels (arrows); in the background, out of focus, yellow tissue full of Leydig cells (between wedges); (**D**) a medium-sized group of dilated tubules close to blood vessels and a brown-yellowish Leydigian area (arrow).

**Figure 8:**
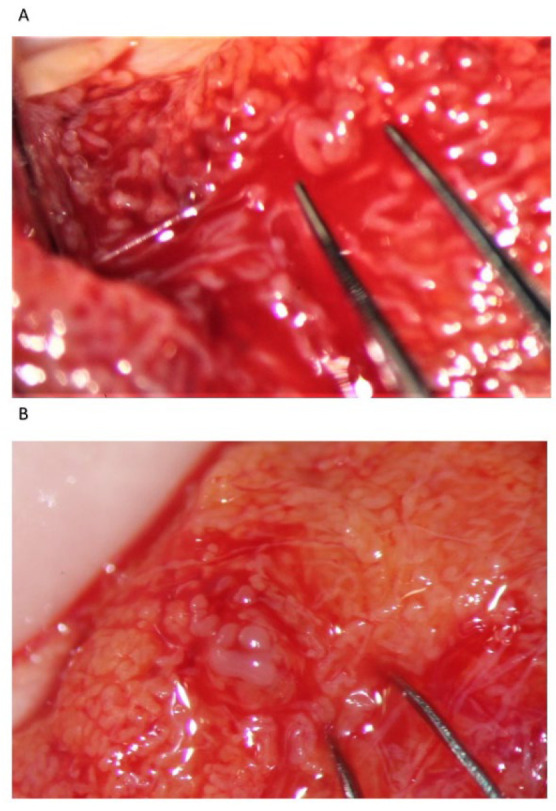
(**A**) An isolated dilated tubule; (**B**) a single dilated tubule surrounded by atrophic tubules.

**Figure 9:**
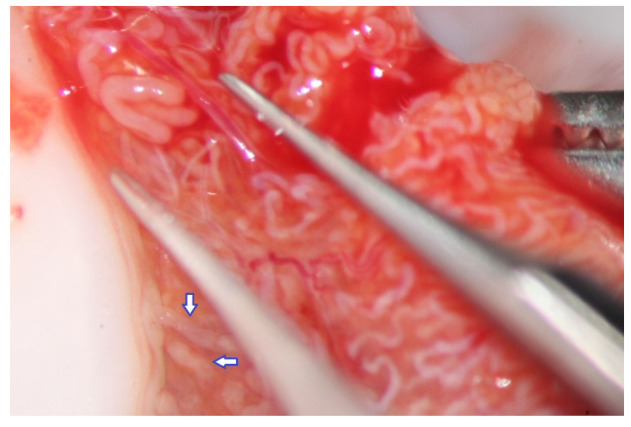
Sometimes dilated tubules appear bloated (between the forceps tips): see the contrast with the slightly less dilated tubules below (arrows).

**Figure 10:**
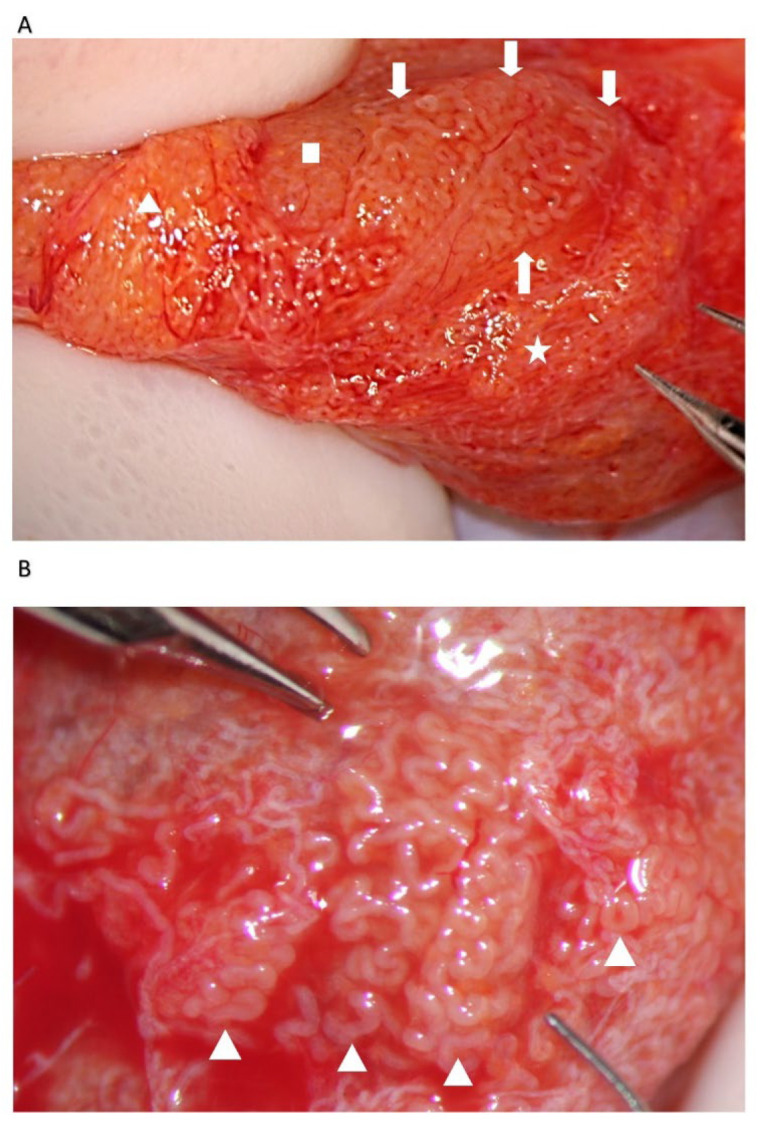
(**A**) Each lobule is made up of tubules of the same caliber, but the caliber decreases from lobule to lobule: dilated (delimited with arrows), less dilated (triangle), narrow (square), and atrophic (star); (**B**) four lobules of dilated tubules emerging from atrophic parenchyma.

Our experience suggests that dilated STs containing sperm may be more easily found close to the small vessel, probably due to the better local blood perfusion, or to clustering Leydig cells ((Figure 4C,D and Figure 7). Sometimes groups of convoluted dilated STs are found (Figure 4) or occupy a small lobule that should be carefully detached and removed (Figure 7); in some cases only dilated segments of otherwise thin STs are found (Figure 8). The best tubules often display a slightly different color than the surrounding ones or may give the impression of being overdistended (Figure 9). The finding of large lobules made of dilated STs is an extremely rare event, at least in our experience (Figure 10).

**Figure 11:**
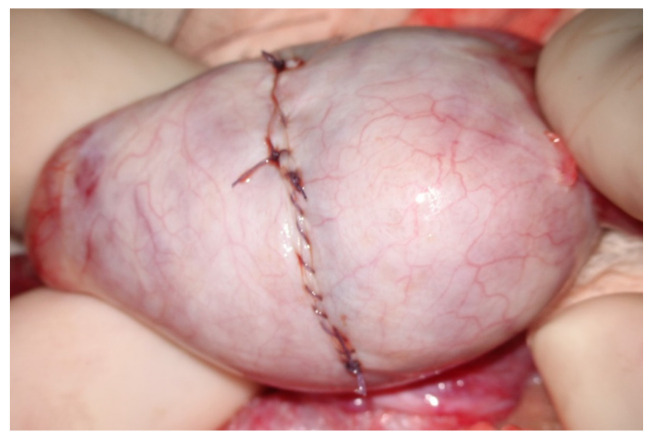
Closure of the albuginea after bivalve opening.

**Figure 12:**
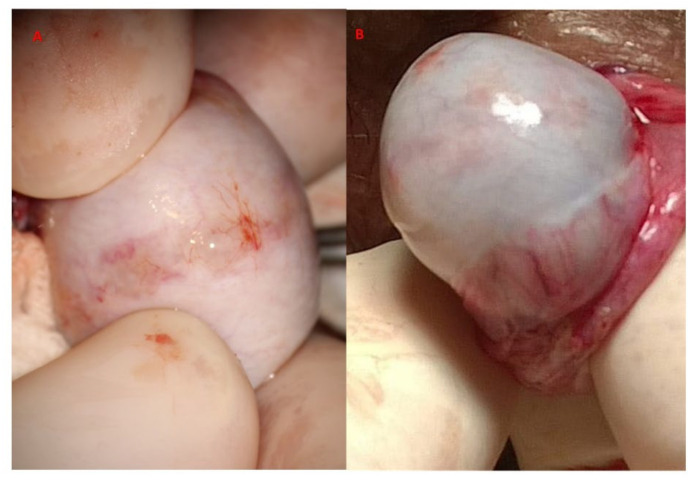
Salvage mTESE after (**A**) one previous failed mTESE (**B**) two previous mTESEs.

At the end of the exploration of the testicular parenchyma at high magnification, the testicular tissue surface is irrigated for antisepsis with Ringer solution (with 80 mg gentamycin/100 mL). Hemostasis is performed when the blood pressure is normalized to avoid postoperative bleeding by gently pressing the testicular tissue for 4 min using gauze wet with the antiseptic solution and eventually (the least possible) using the microsurgical bipolar thermal device. The albuginea incision is closed with a continuous suture of Vicryl 5/0 with a taper-point needle in a running fashion (Figure 11), preferably involving only its external layer to avoid any additional damage to the sub-albugineal vessels. Vicryl is an absorbable suture that does not leave any detectable trace at the testis ultrasound when performed weeks later [22] (Figure 12). Other authors use a 5–0 non-absorbable monofilament suture, such as polydioxanone or a 6–0 nylon suture, to allow the clear identification of the site of incision if a repeat procedure is needed at a subsequent time [19]. The tunica vaginalis opening is repaired by a continuous Vicryl 4/0, after an instillation into the vaginalis cavity of 1 mL saline solution with 2 mg betamethasone, to prevent both pain and tunica vaginalis adhesions, as confirmed in the case of reoperation [23]. The dartos muscle layer and scrotal skin are closed by separate stitches with Vicryl 3/0 suture. MTESE is affected by a minimal blood loss (no more than 3 mL per testis).

**Figure 13:**
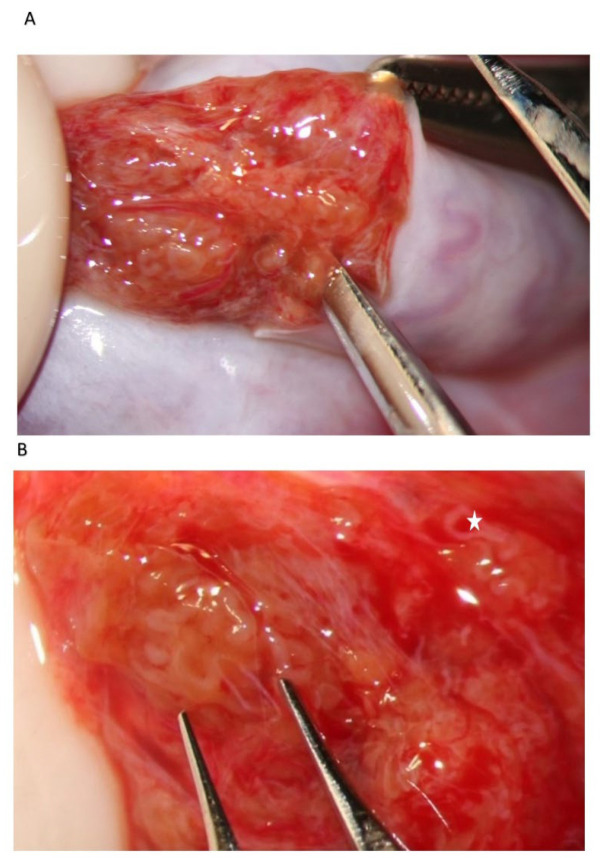
Testis in a patient with Klinefelter syndrome. (**A**) low magnification; (**B**) high magnification (×36): forceps tips indicate a tiny group of dilated tubules inside a brownish area rich with Leydig cells, with atrophic tubules in the background (one atrophic tubule is marked with a star).

The mTESE procedure may proceed slightly differently in particular cases: (i) Incidental testicular lesions may be found in up to 2.9% of patients with NOA [24]. Such lesions are usually benign when their maximum diameter at ultrasound does not exceed 5 mm [25]. Following a preoperative testis ultrasound assessing the intraparenchymal coordinates of the nodule, incision of the albuginea is made to easily reach the nodule, which is completely removed together with a thin layer of intact parenchyma; a frozen section would guide the surgeon to proceed with mTESE in the case of benign lesions (e.g., leydigiomas), or to total orchiectomy, with a consequent search for sperm in the removed testis. (ii) Klinefelter patients may have very small, firm testes. The testicular parenchyma is usually darker, stiffer, and more fragile compared to that of patients with idiopathic NOA. Groups of hyperplastic Leydig cells are dispersed among hyalinized tubules, among which the rare dilated tubules may be found (Figure 13).

## 4. Post-Operative Course

MTESE is a minimally invasive surgery when performed to preserve, as much as possible, the integrity of the testicular parenchyma: a testis ultrasound performed six months after mTESE does not usually reveal any visible scar [22]. Overnight hospitalization is always suggested, particularly for those patients living far away from the hospital. Patients should be examined for a scrotal hematoma prior to discharge. Other prescriptions include oral antibiotics (usually for a week); bed rest and an ice pack to the scrotum for the first 48 h; no scrotal supporters, to avoid testicular retraction in the upper scrotal position; and suture removal ten days after surgery. The post-operative course is usually painless, probably thanks to the betamethasone instillation in the vaginalis tunica and to the careful handling of the spermatic cord; in the case of pain, paracetamol is prescribed for a couple of days. The patient would be able to go back to work in three days, may resume normal sexual activity in ten days, and should wait for twenty days before resuming any intense physical activity.

Complications are extremely rare, particularly when a surgeon with great experience in microsurgery performs an mTESE. In our experience (GMC), intratesticular hematoma occurred once in 1300 procedures, due to hemostasis being performed during uncorrected hypotension after induction of general anesthesia; the testis was opened again, and no signs of testicular damage were seen at ultrasound two weeks and six months later. Complications are more frequently observed when a single or multiple biopsy cTESE is performed [26].

A testosterone follow up assay should be performed 9 and 18 months after surgery as a significant decrease in testosterone serum levels has been described at 3–6 months, with a return to 95% of the baseline testosterone levels at the end of 18 months [26]. Patients whose pre-surgical subnormal testosterone levels have been optimized prior to mTESE should receive testosterone replacement therapy if no further surgery for sperm retrieval is awaited.

## Figures and Tables

**Table 1:** Comparison of the mTESE outcome performed by the same urologist (GMC) in two cohorts of patients with NOA.

	San Paolo Cohort	Procrea Cohort
Years	2004–2009	2015–2017
Number of patients	202	143
Overall sperm retrieval rate (SRR)	80/202 (39.6%)	79/143 (55.2%)
SRR per testis histology subcategories		
Sertoli cells only syndrome	28/125 (22.4%)	45/143 (31.5%)
Maturation arrest	6/16 (37.5%)	11/29 (37.9%)
Hypospermatogenesis	20/26 (76.9%)	27/28 (96.4%)
Focal Sertoli cells only syndrome	26/35 (74.2%)	9/9 (100%)
Hyalinosis	/	2/9 (22.2%)
Intraepithelial neoplasia	/	1/2 (50%)

San Paolo cohort: patients undergoing mTESE at San Paolo Hospital, Milan, Italy. Procrea Cohort: patients undergoing mTESE at Procrea Institute, Lugano, Switzerland.

## Data Availability

Not applicable.
